# First Complete Genome Sequences of Human Sapovirus Strains Classified as GI.3, GI.4, GI.6, GI.7, and GII.7

**DOI:** 10.1128/genomeA.00168-18

**Published:** 2018-03-22

**Authors:** Tomoichiro Oka, Nobuhiro Iritani, Mineyuki Okada, Tomoko Ogawa, Setsuko Iizuka, Chika Tatsumi, Seiya Harada, Kei Haga, Yen Hai Doan

**Affiliations:** a Department of Virology II, National Institute of Infectious Diseases, Tokyo, Japan; b Division of Microbiology, Osaka Institute of Public Health, Osaka, Japan; c Chiba Prefecture Toso Meat Inspection Office, Chiba, Japan; d Division of Virology, Chiba Prefectural Institute of Public Health, Chiba, Japan; e Division of Virology, Shimane Prefectural Institute of Public Health and Environmental Science, Shimane, Japan; f Department of Microbiology, Kumamoto Prefectural Institute of Public Health and Environmental Science, Kumamoto, Japan

## Abstract

We report here the first complete genome sequences of genotype GI.3, GI.4, GI.6, GI.7, and GII.7 sapovirus strains, detected from fecal samples of acute gastroenteritis patients. Complete or nearly complete genome sequences of all 18 genotypes of human sapoviruses are now available for phylogenetic analysis and primer design.

## GENOME ANNOUNCEMENT

Genetically diverse sapovirus (SaV) strains have been detected in fecal specimens from patients with acute gastroenteritis (1–4). We have recently classified human SaV strains into 17 genotypes (i.e., GI.1 to GI.7, GII.1 to GII.7, GIV.1, GV.1, and GV. 2) based on complete major structural protein (VP1) nucleotide sequence (5). Recently, an additional human SaV genotype (i.e., GII.8) has been proposed (6–8). Currently, complete or nearly complete genome sequences of 13 human SaV genotypes (i.e., GI.1, GI.2, GI.5, GII.1, GII.2, GII.3, GII.4, GII.5, GII.6, GII.8, GIV.1, GV.1, and GV.2) are available in public databases. In this study, we determined the first complete genome sequences of genotypes GI.3, GI.4, GI.6, GI.7, and GII.7.

Viral RNA was extracted from fecal suspensions using the High Pure viral RNA kit (Roche) or QIAamp viral RNA minikit (Qiagen). Library preparation from the purified RNA for sequencing on an Illumina MiSeq platform (Illumina) and *de novo* assembly of consensus SaV genome sequence were performed first, as described (9, 10). For the GII.7 SaV strain, we performed seminested long reverse transcriptase PCR (RT-PCR) (amplicon size, 5.7 kb) (10, 11) using the newly designed forward primer 5′-ATGGCTTCYAAGCCATTCTACC-3′, which corresponds to the 22 nucleotides (nt) from the predicted start codon of the open reading frame (ORF) 1 of genotype GII.1 (GenBank accession no. AJ249939 and AY237419), GII.2 **(**AY237420), GII.3 **(**AY603425), GII.5 (LC190463), and GII.6 (AY646855) SaV genomes in combination with two primers designed in the previously determined VP1-encoding-region sequence (12). Furthermore, the 3′ ends of SaV genomes of the GI.4, GI.7, and GII.7 SaV strains (∼2.5 kb) were amplified by RT-PCR with a gene-specific forward primer and the reverse primer TX30SXN, which was complementary to the 3′-end poly(A) tail (9–11). The 5′ terminal nucleotide sequence of the five SaV strains was further determined by seminested PCR-based 5′-RACE (rapid amplification of cDNA ends) (∼0.5 kb) (9, 10). These PCR products were purified and sequenced directly and/or after cloning using the BigDye Terminator Cycle sequence kit v3.1 and the 3130 Genetic Analyzer capillary sequencer (Applied Biosystems), or library preparation (from the purified PCR product) and an Illumina MiSeq sequencer (10). The full-length genome sequences of the genotype GI.3, GI.4, GI.6, GI.7, and GII.7 SaV strains were assembled using the Sequencher program v4.10.1 (GeneCodes) and analyzed with Genetyx-Mac software v16.0.4 (Genetyx Corporation).

The genomes of GI.3 Hu/OH08021/2008/JP, GI.4 Hu/SV/Chiba/000496/2000, GI.6 Hu/SV/Chiba/000764/2000, GI.7 Hu/D1714-B/2008/JPN, and GII.7 Hu/20072248/2008/JP SaV strains consist of 7,442, 7,436, 7,443, 7,452, and 7,462 nt, respectively, excluding the poly(A) tail. All of these SaV genomes were predicted to encode two ORFs, a short 5′ untranslated region (UTR) (12 or 13 nt long) and a 3′-UTR (78 to 112 nt long). The 5′ terminal sequence was conserved as GTG, similarly to those of other SaVs (10, 11).

The new sequence data determined in this study will be useful in designing more broadly reactive primers and probes for human SaV detection PCR, as well as establishment of a nonstructural protein coding region typing scheme like that recently established for norovirus (13).

### Accession number(s).

The genome sequences of GI.3 Gu/OH08021/2008/JP, GI.4 Hu/SV/Chiba/000496/2000, GI.6 Hu/SV/Chiba/000764/2000, GI.7 Hu/D1714-B/2008/JPN, and GII.7 Hu/20072248/2008/JP SaV strains have been deposited in GenBank under the accession no. AB623037, AJ606693, AJ606694, AB522390, and AB630067, respectively.
